# Introduction

**DOI:** 10.1054/bjoc.2001.1745

**Published:** 2001-04

**Authors:** J Glaspy

**Affiliations:** UCLA School of Medicine, 200 Medical Plaza, Suite 120, Los Angeles, CA 90024, USA


*British Journal of Cancer* (2001) **84** (Supplement 1), 1–2
© 2001 Cancer Research Campaign
doi: 10.1054/ bjoc.2001.1745, available online at http://www.idealibrary.com on IDEAL®

### Introduction

**J Glaspy**

UCLA School of Medicine, 200 Medical Plaza, Suite 120, Los Angeles, CA 90024, USA

We live in exciting times. The last decade has seen a shift in emphasis in medical practice toward an appropriate focus on optimizing patient functional status and quality of life. In the field of oncology, this trend has been associated with dramatic improvements in anti-emesis, myelopoietic support, and the recent recognition that degrees of anaemia previously assumed to be unimportant seriously impact function and require attention. These advances continue to improve the lives of cancer patients. As biotechnology advances, the potential for further symptomatic improvement for patients is indeed great.

The recognition over the last 4 years that treatment of mild and moderate degrees of anaemia with recombinant human erythropoietin (rHuEPO) in patients with serious chronic medical conditions such as cancer is associated with clinically significant improvements in energy, activity and overall quality of life has been a watershed event in our understanding of optimal palliative management. I regard this as one of the most significant developments of the last decade in terms of impact on patient care. Still, our understanding of the optimal approach to erythropoietic management of these patients is incomplete and a better understanding of several key issues is required, including the relationship of dose to response rate; the relationship of dose to rapidity of response; the effect of iron supplementation on response and the impact of treatment on the efficacy of radiotherapy and of chemotherapy.

This special edition of *British Journal of Cancer* is devoted to darbepoetin alfa, a novel erythropoiesis stimulating protein (NESP), and includes articles describing its preclinical development, as well as preliminary data from clinical trials of NESP in patients with cancer.

Darbepoetin alfa (ARANESP™, Amgen Inc, Thousand Oaks, CA) is the first of a new generation of erythropoiesis stimulating proteins. It stimulates erythropoiesis by binding to the human erythropoietin receptor, just as rHuEPO does. NESP, however, is biochemically distinct from rHuEPO, containing additional sialic acid, which prolongs its serum half-life and thus increases its in vivo activity (Egrie et al, 1997).

In the preclinical studies described by Egrie and Browne, NESP was shown to have a serum half-life approximately 3-fold longer than rHuEPO, resulting in increased in vivo biological activity (Egrie and Browne, 2001). The longer half-life and increased biological activity suggested that less frequent dosing was likely, enhancing the potential for growth factor administration to be synchronized with chemotherapy cycles.

The longer half-life suggested by these preclinical studies was confirmed when NESP was tested in human patients. In pharmacokinetic studies involving patients with renal disease, NESP was shown to have a 3-fold longer terminal half-life than rHuEPO following intravenous administration (mean = 25.3 versus 8.5 hours, $P = 0.0008$) (Macdougall et al, 1999). Subcutaneous administration extended the half-life to 48.8 hours. Furthermore, NESP was shown to have the same excellent safety and tolerability as rHuEPO.

NESP license application for treatment of renal anaemia has been filed worldwide, following extensive clinical trials demonstrating the benefits of the drug in this patient group. The agent is now undergoing clinical trials in patients with cancer. The preliminary results of three of these studies are reported in this special edition of *British Journal of Cancer*.

In this edition, Heatherington et al confirm the extended half-life of NESP in their pharmacokinetic study of anaemic patients receiving concurrent chemotherapy (Heatherington et al, 2001). A mean terminal half-life of 32.6 hours was demonstrated after subcutaneous administration. Weekly dosing of NESP and a dose–response relationship were examined in the dose-escalation trial by Smith et al (Smith et al, 2001). In this trial, 61–83% of patients with chronic anaemia of cancer who were not receiving chemotherapy responded (increase in haemoglobin ≥ 2.0 g $dl^{-1}$) to once-weekly subcutaneous administration of 1.0–4.5 mcg $kg^{-1}$ NESP.

In a subset of a larger trial, in which 107 patients received concurrent chemotherapy, Glaspy et al confirmed the feasibility of reduced-frequency dosing, and showed a dose-dependent relationship between NESP and multiple measures of efficacy, including proportion of patients responding to NESP and change in haemoglobin (Glaspy et al, 2001). NESP was well tolerated in all trials, and there was no evidence of antibody formation.

The final paper in this edition, by Demetri, discusses some of the current challenges in the management of anaemic cancer patients and considers the future role of NESP in this setting (Demetri, 2001). Taken in aggregate, these articles suggest that there may be dose–response and dose-rapidity of response relationships with NESP, which may enhance our ability to optimally palliate patients. They also point to opportunities for future investigations.

### REFERENCES

Demetri GD (2001) Anaemia and its functional consequences in cancer patients: current challenges in management and prospects for improving therapy. *Br J Cancer* **84** (Supp 1): 31–37

Egrie J and Browne J (2001) Development and characterization of novel erythropoiesis stimulating protein (NESP). *Br J Cancer* **84** (Supp 1): 3–10

*Correspondence to:* Dr John Glaspy

Egrie JC, Dwyer E, Lykos M, Hitz A and Browne JK (1997) Novel erythropoiesis stimulating protein (NESP) has a longer serum half-life and greater in vivo biological activity than recombinant human erythropoietin (rHuEPO). *Blood* **90**: 56a (abstract 243)

Glaspy J, Jadeja JS, Justice G, Kessler J, Richards D, Schwartzberg L, Rigas J, Kuter D, Harmon D, Prow D, Demetri G, Gordon D, Arseneau J, Saven A, Hynes H, Boccia R, O'Byrne J and Colowick AB (2001) A dose-finding and safety study of novel erythropoiesis stimulating protein (NESP) for the treatment of anaemia in patients receiving multicycle chemotherapy. *Br J Cancer* **84** (Supp 1): 17–23

Heatherington AC, Schuller J and Mercer AJ (2001) Pharmacokinetics of novel erythropoiesis stimulating protein (NESP) in cancer patients: preliminary report. *Br J Cancer* **84** (Supp 1): 11–16

Macdougall IC, Gray SJ, Elston O, Breen C, Jenkins B, Browne J and Egrie J (1999) Pharmacokinetics of novel erythropoiesis stimulating protein compared with epoetin alfa in dialysis patients. *J Am Soc Nephrol* **10**: 2392–2395

Smith RE Jr, Jaiyesimi IA, Meza LA, Tchekmedyian NS, Chan D, Griffith H, Brosman S, Bukowski R, Murdock M, Rarick M, Saven A, Colowick AB, Fleishman A, Gayko U and Glaspy J (2001) Novel erythropoiesis stimulating protein (NESP) for the treatment of anaemia of chronic disease associated with cancer. *Br J Cancer* **84** (Supp 1): 24–30

*© 2001 Cancer Research Campaign*

## References

[PDF_00088] Demetri G. D. (2001). Anaemia and its functional consequences in cancer patients: current challenges in management and prospects for improving therapy.. Br J Cancer.

[PDF_00091] Egrie J. C., Browne J. K. (2001). Development and characterization of novel erythropoiesis stimulating protein (NESP).. Br J Cancer.

[PDF_00105] Glaspy J., Jadeja J. S., Justice G., Kessler J., Richards D., Schwartzberg L., Rigas J., Kuter D., Harmon D., Prow D. (2001). A dose-finding and safety study of novel erythropoiesis stimulating protein (NESP) for the treatment of anaemia in patients receiving multicycle chemotherapy.. Br J Cancer.

[PDF_00111] Heatherington A. C., Schuller J., Mercer A. J. (2001). Pharmacokinetics of novel erythropoiesis stimulating protein (NESP) in cancer patients: preliminary report.. Br J Cancer.

[PDF_00114] Macdougall I. C., Gray S. J., Elston O., Breen C., Jenkins B., Browne J., Egrie J. (1999). Pharmacokinetics of novel erythropoiesis stimulating protein compared with epoetin alfa in dialysis patients.. J Am Soc Nephrol.

[PDF_00118] Smith R. E., Jaiyesimi I. A., Meza L. A., Tchekmedyian N. S., Chan D., Griffith H., Brosman S., Bukowski R., Murdoch M., Rarick M. (2001). Novel erythropoiesis stimulating protein (NESP) for the treatment of anaemia of chronic disease associated with cancer.. Br J Cancer.

